# Research domain criteria: a final paradigm for psychiatry?

**DOI:** 10.3389/fnhum.2015.00488

**Published:** 2015-09-08

**Authors:** Walter Glannon

**Affiliations:** Department of Philosophy/Arts, University of CalgaryCalgary, AB, Canada

**Keywords:** biomarkers, genetics, neuroimaging, neuro-immune interactions, neuromodulation, psychiatric disorders, Research Domain Criteria (RDoC)

## Introduction

Since the US Congress designated the 1990s as the “Decade of the Brain,” much of the research in psychiatry has endeavored to explain psychiatric disorders in terms of interconnected sets of dysfunctional neural circuits. The shift in focus from behavioral manifestations to the underlying neurobiology of mental illness has driven the field of biological psychiatry in general and the Research Domain Criteria (RDoC) in particular. The aim of the RDoC is to identify brain mechanisms that can explain the etiology and pathophysiology of psychiatric disorders, provide earlier and more accurate diagnosis, and predict treatment responses and outcomes (Insel et al., 2010; Casey et al., 2013). It incorporates genetics and behavioral science, including the influence of the environment on neurodevelopment, into a broad neuroscientific paradigm of psychiatry. By exploring the causes of mental illnesses and how these can inform interventions to modulate neural pathways, the circuit-based RDoC offers a more satisfactory account of these illnesses than the symptom-based Diagnostic and Statistical Manual of Mental Disorders (DSM-5). In its current form, however, the RDoC may be too limited a theoretical model to provide a complete understanding of why mental illness develops, how it progresses, and how different treatments might control it. This raises the question of whether a more comprehensive version of the RDoC, or a different paradigm altogether, will be needed to guide research and clinical practice in psychiatry.

## History

Past attempts to conceptualize a sound paradigm for psychiatry proved elusive because of limited knowledge of how brain function enables the mind and how brain dysfunction disables it. Although many practitioners of psychoanalysis separate the psyche from the brain, the father of psychoanalysis, Freud, attempted to formulate a scientific psychology of the mind in his *Project for a Scientific Psychology* (Freud, 1895). This attempt was unsuccessful because at the time little was known about the neurobiological underpinning of the psyche. The notorious first era of psychosurgery developed and practiced by Moniz and Lima in the 1930s and 1940s and Freeman and Watts in the 1940s and 1950s was based on the idea that psychiatric disorders were caused by abnormalities in white-matter tracts in the prefrontal cortex and their projections to limbic structures (Pressman, 1998). Creating lesions in these tracts and severing connections in neural pathways to relieve symptoms was the ill-conceived rationale for Moniz and Lima's prefrontal leucotomy and Freeman and Watts' frontal lobotomy. These procedures failed to achieve their therapeutic goal and often resulted in significant cognitive, affective and motor impairment in many patients. The advent of antidepressant and antipsychotic drugs in the 1950s obviated the need for psychosurgery. This pharmacological approach to treating depression was based on a monoamine hypothesis, which explained mood disorders in terms of abnormalities in mechanisms regulating dopamine, adrenaline, noradrenaline and serotonin. Treating schizophrenia was based more specifically on a dopamine hypothesis, and later a glutamate hypothesis, regarding abnormalities in these neurotransmitters. While these hypotheses are consistent with the RDoC in offering a neurobiological explanation of psychiatric disorders, they are incomplete because they do not explain their etiology or predict treatment outcomes. The RDoC's inclusion of genetic and other factors that influence neurotransmitters and neural circuits more generally provides a more adequately informed model than previous ones to account for mental illness.

## Biomarkers, neuromodulation, and neuroimaging

There are at least three respects in which the brain-systems focus of the RDoC is a major advance in understanding and treating psychiatric disorders: biomarkers; neuromodulation; and neuroimaging that detects these signatures and guides these techniques. Biomarkers may be mutations detected through genetic testing and screening, abnormal proteins discovered in bodily fluids, or structural and functional features of the brain displayed in neuroimaging (Boksa, 2013). Detecting these biological signatures might enable researchers and clinicians to identify those at risk of developing a disorder when they are in a pre-clinical state or have prodromal symptoms. In schizophrenia, identifying a biomarker such as cerebral white matter abnormalities when a person is experiencing pre-psychotic positive symptoms such as mild hallucinations and paranoia but before a first psychotic episode could warrant early intervention (Lieberman et al., 2013). This could minimize adverse changes in the brain and reduce the chronic severity of the illness. In addition, biomarkers could contribute to more accurate diagnosis and prognosis of this and other conditions when symptoms alone are ambiguous. Currently, the most promising area of biomarker research in psychiatry is predicting responses to different treatments. In a recent study involving patients with major depressive disorder (MDD), hypometabolism in the insula displayed by functional neuroimaging (PET) was associated with a positive response to cognitive-behavioral therapy (CBT) in symptom reduction but a poor response to the SSRI escitalopram (McGrath et al., 2013). In contrast, insula hypermetabolism was associated with a positive response to the drug and a poor response to CBT. This study showed that a treatment-specific biomarker could guide treatment selection for patients with MDD.

Another significant development in circuit-based psychiatry has been the use of electrical current in deep brain stimulation (DBS) and light in optogenetics to activate or inhibit neural activity (Deisseroth et al., 2015). DBS is especially valuable because it can both probe dysfunctional circuits in real-time and modulate them in treatment-refractory depression and obsessive-compulsive disorder (Lozano and Lipsman, 2013). MRI-guided stereotactic functional neurosurgery for these disorders is more effective and safer than the crude and often harmful psychosurgery of the past. Biomarkers, neuromodulation and neuroimaging together may lead to more personalized diagnosis and treatment for psychiatric patients. They could contribute to “precision medicine” for psychiatry, where “treatments are targeted to the needs of individual patients on the basis of genetic, biomarker, phenotypic, or psychosocial characteristics that distinguish a given patient from other patients with similar clinical presentations” (Jameson and Longo, 2015, p. 1).

Explaining psychiatric disorders in terms of dysfunctional circuits in the brain also allows for alternative novel hypotheses that go beyond monoamines and dopamine. Some of these are based on findings of abnormalities in the brain's resting-state activity. Dehaene and Changeux (2011) have hypothesized that schizophrenia is a disorder of consciousness caused by disrupted information integration in the brain. Diffusion tensor imaging of individuals with positive symptoms of schizophrenia supports this hypothesis by revealing impaired connections between prefrontal and posterior regions of the cortex, hippocampus and thalamus, a distributed network that mediates conscious processing (Kubicki et al., 2005). Similarly, Northoff (2014, Ch. 27) has noted abnormalities in resting-state activity in both schizophrenia and depression displayed by imaging. In schizophrenia, there is impaired functional connectivity and low-frequency fluctuations in midline regions. In depression, there is hyperactivity in the midline network and hypoactivity in the lateral network. This imbalance disrupts the connection between the brain and the environment, with a pathological increase in self-focus and a corresponding decrease in environment-focus at the phenomenal level of consciousness.

Genetics might also help to explain some psychiatric disorders as disorders of memory content. Generalized anxiety and posttraumatic stress disorder (PTSD), for example, can be described as conditions in which the representation of a disturbing or traumatic memory of an event persists in the brain to the point of becoming maladaptive or pathological (Schacter, 2001, Ch. 7). Why some people rather than others develop these conditions may be partly explained by differences in transcription factors such as cyclic AMP response element-binding protein (CREB) and how these factors regulate the formation and storage of episodic memories. In addition, differences in how genes code for dopamine and regulate its release in the brain could account for differences in placebo responses among patients (Hall et al., 2015). This is another respect in which brain-based mechanisms regulating cognitive and affective states can contribute to precision medicine in diagnosing and treating psychiatric disorders, where placebo responses are common. By accounting for the variability of placebo responses among patients with depression, anxiety and other conditions, these mechanisms could improve therapy by inducing these responses as additive to the salutary effects of pharmacological agents.

## Limitations

The neural circuits on which the RDoC focuses may not adequately consider the effects of neuro-immune and neuro-endocrine interactions in the pathophysiology of depression, schizophrenia and possibly other disorders. Cytokines released in response to infection may result in elevated levels of inflammatory biomarkers in the blood. These in turn may lead to inflammatory changes in the brain and alteration of neural circuits that could trigger or exacerbate mood disturbances and cognitive and volitional impairment in depression (Raison et al., 2006, 2013). As another example of neuro-immune interaction, the neuropathology in some psychiatric disorders may be traced to prenatal events. Maternal antibodies produced in response to infection during pregnancy can activate immune processes that can trigger an inflammatory reaction in the fetal brain and alter its development (Patterson, 2011). There is some association between this type of reaction and the risk of schizophrenia and autism in offspring. These and other adverse processes in the maternal-fetal environment may be part of an explanation for abnormalities in synaptic pruning, which has been suggested as one response to the question of why many neurodevelopmental disorders emerge during adolescence (Paus et al., 2008). The extent to which maternal antibodies are present during fetal gestation and cause an inflammatory reaction in the fetus might also partly account for behavioral and other phenotypic differences among individuals affected by schizophrenia. Neuro-immune interactions during the prenatal period underscore the importance of prenatal care and how more biologically informed reproductive choices might prevent some forms of mental illness. Another limitation of circuit-level explanations is that they cannot account for how psychosocial factors influence people's stress responses to environmental stimuli or the variability of these responses. Chronic stress can induce hyperactivity in the amygdala fear system and impair the reward system in causing depressive symptoms. While biological psychiatry can explain this process in neurobiological terms, it cannot explain the causal role of an affected person's mental states in the etiology of depression. Nor can circuit-level explanations of this and other psychiatric disorders account for the phenomenology of experiencing delusions, hallucinations, avolition, anhedonia, and low or elevated mood. Yet the subjective aspect of these disorders is essential to knowing how people are affected by them and validating symptom relief from different treatments because they are diseases of dysfunctional brain-mind and mind-brain interaction. Although the identification of more psychiatric biomarkers through genetic testing and neuroimaging will contribute to a better understanding of psychiatric disorders, it will not offer a complete explanation of why people develop these disorders or why some respond more favorably to treatment than others. Because of the multifactorial etiology of depression and other conditions and the heterogeneity of expression of their symptoms, examining them in terms of neural circuits will not replace but supplement and refine other criteria for predicting, diagnosing, and monitoring responses to interventions to control and possibly prevent them.

## Conclusion

Psychiatric disorders result from interaction among neurons, genes, immune and endocrine systems and the affected person's psychological response to the natural and social environment. Explaining these disorders at a brain-systems level is necessary but not sufficient to understand how they develop and how they can be treated with pharmacological and behavioral therapies targeted to individuals affected by them. Ordered and disordered states of brain and mind are influenced by factors both inside and outside of the brain. Mental illness currently constitutes 7.4% of the global burden of disease, and its incidence will increase exponentially in the future (Becker and Kleinman, 2013). The World Health Organization (2012) estimates that depression will be the leading cause of global disease burden by 2030. Despite its limitations, RDoC offers the most conceptually coherent and scientifically sound paradigm for explaining psychiatric disorders. Still, a more comprehensive version of the RDoC that includes processes in addition to circuit-level mechanisms will put psychiatry in a better position to meet the challenges posed by mental illness. This is not a novel model but a further stage of an evolving non-reductive understanding of mental illness based on complex interactions among neurobiological, psychological and social factors. Such a model will enable researchers and clinicians to more effectively relieve the burden of psychiatric disorders and improve the quality of life for the millions of patients suffering from these diseases of the brain and mind.

### Conflict of interest statement

The author declares that the research was conducted in the absence of any commercial or financial relationships that could be construed as a potential conflict of interest.
